# The Effectiveness of Daily Mindful Breathing Practices on Test Anxiety of Students

**DOI:** 10.1371/journal.pone.0164822

**Published:** 2016-10-20

**Authors:** Hyunju Cho, Seokjin Ryu, Jeeae Noh, Jongsun Lee

**Affiliations:** 1 Department of Psychology, Yeungnam University, Gyeongsan-si, South of Korea; 2 Department of Psychology, Kangwon National University, Chuncheon-si, South of Korea; Kyoto University, JAPAN

## Abstract

The present study examined the effectiveness of daily mindful breathing practices on test anxiety of university students. A total of 36 participants were randomly assigned to one of three conditions: a training mindful breathing condition (n = 12), a training cognitive reappraisal condition (contrast group, n = 12), and a non-training condition (control group, n = 12). Each of the participants trained by themselves for 6 days after they had taken one session of education for mindful or cognitive reappraisal practices. They wrote their experiences on daily worksheets and sent it by mobile with taking pictures that were confirmed by the researcher. Before and after training, each of the participants completed a questionnaire to assess: test anxiety, positive thought, and positive affect. The results of the study showed that both mindful breathing practice and cognitive reappraisal practice yielded large effect sizes in reducing test anxiety. In addition, the mindful breathing condition scored significantly higher on positive thoughts than the cognitive reappraisal and control conditions. The findings of this study suggest that both daily mindful breathing and cognitive reappraisal practices were effective in reducing test anxiety; however, mindful breathing increased positive automatic thoughts to a greater extent than cognitive reappraisal.

## Introduction

Test taking begins in childhood and extends to late adulthood. Even if a child develops significant anxiety surrounding tests, they are compulsory, and this can lead to a great deal of distress. Test anxiety has a negative impact on learning, is a major cause for underachievement, and prevents some students from reaching their academic potential [1]. In addition, it has been reported that test anxiety caused students to experience impaired emotional working memory capacity [2], which is correlated negatively with test performance [3]. Especially, 20% to 40% of students suffered from examination-related anxiety [4,5]. If people with test anxiety are not able to effectively deal with this problem, their academic work suffers and they are at risk for developing anxiety disorders. Thus, students who have high levels of test anxiety need to prevent themselves from developing anxiety disorders. There has been reported a little of evidence-based therapy in reducing test anxiety, even though test anxiety can impair deeply academic performance and well-being. According to cognitive models of anxiety and performance [6], test anxious individuals show impaired performance because they are too preoccupied with the thoughts about failure before or during a specific evaluation. Previous studies showed that cognitive therapy which focuses on a person’s negative beliefs (using cognitive reappraisal) is an effective way of on relieving test anxiety [7,8]. More specifically, cognitive therapy that focused on the dysfunctional cognitive processes of current or future experiences appeared to be effective in reducing test anxiety for university students, especially for introverted students as compared with extroverted students [7].

On the other hand, Mindfulness-Based Intervention (MBI), such as Mindfulness-Based Stress Reduction(MBSR)[9] which has been shown to be effective in reducing pain, stress, anxiety, negative affect and depression [10,11], has come into the spotlight. Mindfulness teaches participants to pay attention to their internal experiences, such as breath, body sensation, emotion and cognition. It further teaches them to observe these experiences without judgment [9]. People practicing mindfulness strive to stay in contact with the experience of the present moment which may lead to: relaxation, cognitive change, acceptance and self-regulation [12]. Furthermore, it leads to changes in values clarification, cognitive and behavioral flexibility [13]. Also, paying attention to anxiety-related sensations and thoughts can lead to reductions in the emotional reactivity typically elicited by anxiety symptoms [14]. The practice of mindfulness skills may improve people’s ability to tolerate negative emotions and their ability to deal with them effectively [15].

However, it is suggested that test anxiety is not a disorder, but rather is a subjective emotional state [16]. As such, it does not thus far require a therapeutic program or treatment with a professional therapist, nor does it require much time to effectively reduce test anxiety. There is evidence that three weekly mindfulness training sessions with university students was enough to be effective at reducing symptoms of anxiety and stress [17]. In addition, Burg, Wolf and Michalak [18] found that participants who were better able to self-regulate their attention to breathing during 5 minutes of the mindful breathing exercises (MBE) displayed significantly higher values on two of three indices of HRV (Heart Rate Variability). The authors speculated that higher HRV during brief breathing mindfulness might be at least be due in part to an indication of enhanced self-regulation about their negative thinking and emotion. The aforementioned studies suggest that even one brief mindfulness intervention session might help anxious individuals with their negative thoughts and anxious symptoms. However from a neurological perspective, Linda and Hanson [19] have argued that *repeated experiences* help people to form stronger synaptic connections and rewire internal conditioned responses in the brain toward stressors. This suggests that repetition by means of continued practice is essential for an anxious person to develop alternative emotions and thoughts. Therefore, we designed our study using repeated practice sessions during a period of one week instead of using only a single practice session for highly anxious students. Also, we selected cognitive reappraisal practices as a contrast condition because it can be comparable to mindful practices in terms of effectiveness of intervention on test anxiety.

In summary, this study examined the efficacy of daily mindful breathing practices in reducing test anxiety, automatic thoughts, and mood. We expected that mindfulness breathing practices would show increased positive thinking and positive affect, and decreased test anxiety, as compared to cognitive reappraisal practice and a non-training control condition.

## Materials and Methods

### Participants and Ethics statements

Students (N = 233) who were taking psychology classes at the Yeungnam University in South Korea participated in this study. All participants were given a brief explanation about the study with written consent form, followed by a scale of test anxiety at the end of the class. Information regarding a small compensation (gift coupon) for the study was provided at the screening stage. Participants were also informed that the aim of the study was to investigate the relationship between test anxiety and daily mood. Thirty- six highly anxious individuals were invited to the present study. Participants were screened using the upper 30^th^ percentile of total scores of test anxiety because 20% to 40% of students suffered from examination-related anxiety [4,5]. The mean age of participants was 20.1(SD = 1.47) years, and 58.3% (N = 21) were female and 41.7% (N = 15) were male. The study procedures were reviewed and approved by IRB (Institutional Review Board of Yeungnam University; 7002016A2015001).

### Procedures

**Fig 1:**
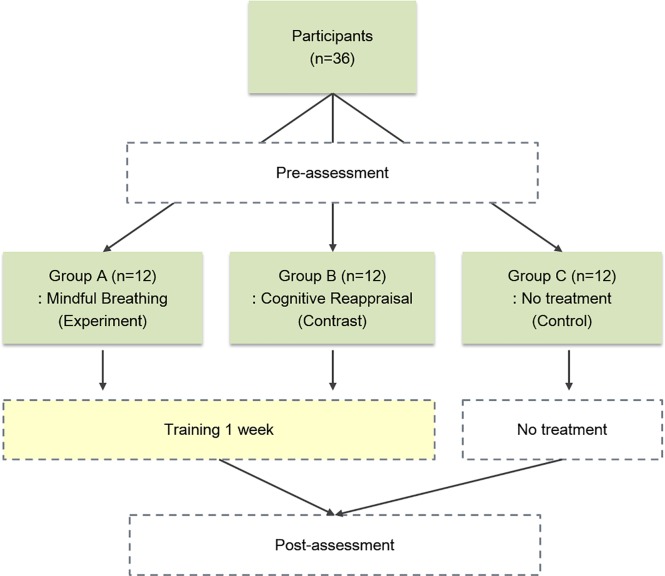
Process of the current study.

Participants attended the experiment on average within 1 week after they had completed the test anxiety questionnaire at the screening stage. On arrival, they were given an information sheet and had an opportunity to ask questions about the study, followed by a written consent form. Using an Excel program, participants were allocated to one of three groups: the MBP, the CRP and a non-training control. They then completed a series of pre-training measures: ATQ-P and PANAS. After completing baseline measures, participants of the MBP completed a mindfulness breathing session while those of the CRP had a cognitive reappraisal session. Each training technique was explained by the licensed clinical psychologist at the first session. At the end of the 1^st^ session, the experimenter checked whether the participant understood their assigned training technique, and had a boost session if necessary. Both trainings were provided individually. The participants of both groups (MBP & CRP) were asked to perform 30 minutes of practice daily for the next week. They were also asked to complete their experiences during the session on the daily worksheet. The participants took a photo of a daily worksheet and sent it to the experimenter using a mobile phone. The experimenter provided feedback on a daily basis in order to encourage them to adhere to their routine. After completing 7 sessions (1 session at the experiment room and 6 sessions at home), participants of both groups visited the experiment room and completed post-training measures: RTA, ATQ-P, PANAS. The control group completed pre-and post-training measures on the 1^st^ day and 8^th^ day, respectively. All participants were given a debriefing about the study and a gift coupon on the 8^th^ day. There were no dropouts. After the completion of the experiment, the control group was provided with an instructional session on mindfulness breathing technique and practices. The final session of the experiment was on average 2 weeks prior to the participants’ final exam. A flow chart of this study is presented in Fig 1.

### Measures

#### Test anxiety

The Revised Test Anxiety (RTA; [20]) was used to screen and assess participants for their level of anxiety. It contains 20 items. The RTA had high internal consistency in the current study (Chronbach’s alphas was .73 in pre-assessment and .91 in post-assessment).

#### Positive thoughts

The Automatic Thoughts Questionnaire-Positive (ATQ-P; [21]) was used to measure automatic thoughts, which contained 30-items. Participants were instructed to select a number ranging from 1(not at all) to 5(very much so) for each item to indicate how often they feel/experience the item described (e.g., “I am respected by my peers”, “I’m fun to be with”). The ATQ-P had a high internal consistency in the current study (Chronbach’s alphas was .91 in pre-assessment and .92 in post-assessment).

#### Positive affect

The Positive And Negative Affect Schedule (PANAS; [22]), was used to measure affect. The PANAS contained a total of 20 items that included 10 items measuring positive affect (e.g., excited, enthusiastic) and 10 items measuring negative affect (e.g., being distressed, upset). However, we used only positive affect items of PANAS. Participants were instructed to select a number ranging from 1(Not at all) to 5(Very much so) for each item. Internal consistency of this study was Chronbach’s alphas = .65 at pre-assessment and .76 at post-assessment.

### Data analysis

Data was analyzed using Statistical Package for Social Sciences (SPSS) 18.0. Baseline differences of groups were evaluated with a chi-square test and one-way analysis of variance (ANOVA). As there were no significant differences on age, gender and baseline measures, a 3 (Condition: MBP, CRP, Control) x 2 (Time: pre-training, post-training) repeated measures MANOVA were performed, followed by repeated measures univariate analyses of variance (ANOVA).

## Results

### Sample Characteristics

A visual inspection of their histograms, normal Q-Q plots and box plots showed that the measures were approximately normally distributed among groups, with a skewness range from -.82 to 1.05 and a kurtosis range from -.86 to .98 for the MBP, a skewness range from -.87 to 1.07 and a kurtosis range from -1.54 to 1.72 for the CRP, and a skewness range from -1.07 to 1.80 and a kurtosis range from -1.03 to 4.44 for the control group [23,24,25,26]. A Levene’s test verified the equality of variances in the sample (homogeneity of variance) (*p* > .05) [27].

### Group Equivalence

We analyzed the participants using several measures (i.e., gender, age, RTA, PANAS and ATQ-P) at pre-assessment for group equivalence. No between group differences were apparent in gender, χ^2^ (2, N = 36) = 2.06, p = .36, and age (*F* [2, 33] = .98, *p* = .39). Furthermore, the groups did not differ on RTA score, F [2, 33] = .51, *p* = .61 or ATQ-P score, F [2, 33] = .08, *p* = .92 and PANAS score, *F* [2, 33] = .57, *p* = .57.

### Effectiveness of MBP and CRP on the outcome measures

**Fig 2:**
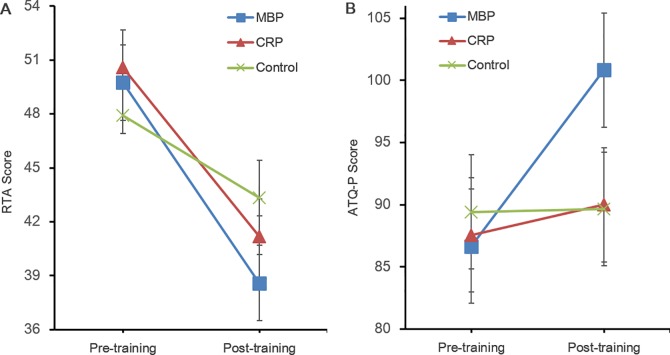
Changes of the RTA and ATQ-P among groups between pre- and post- training. MBP = Mindful Breathing Practice; CRP = Cognitive Reappraisal Practice; RTA = Revised Test Anxiety scale; ATQ-P = Automatic Thoughts Questionnaire-Positive scale.

To investigate the effectiveness of the practice on participants’ level of test anxiety and other measures (ATQ-P, PANAS), 3(Groups: MBP vs. CPR vs. control group) x 2 (Time: pre-assessment, post-assessment) repeated measures MANOVA was performed. The results are presented in Table 1. The analysis revealed a significant Group x Time interaction, Wilks’ Δ = .63, *F* (6, 62) = 2.69, *p* < .05, *η*_p_^2^ = .21, and a significant main effect of Time, Wilks’ Δ = .29, *F* (3, 31) = 25.40, *p* < .001, *η*_p_^2^ = .71. The main effect of Group was not observed, Wilks’ Δ = .95, *F* (6, 62) = .27, *p* = .95, *η*_p_^2^ = .03. Univariate tests were then conducted for each dependent measure. For the RTA, the main effect of Time was significant, *F* (1, 33) = 57.05, *p* < .001, *η*_p_^2^ = .63, and Group x Time interaction showed a trend, *F* (2, 33) = 3.14, *p* = .056, *η*_p_^2^ = .16. Further paired sample t tests revealed that all groups showed a significant decrease in the RTA over time. However, the effect sizes of both groups MBP and, CRP appeared to be higher than those of the control group (MBP: *d* = 1.42; CRP: *d* = 1.23; Control: *d* = 0.56). For the ATQ-P, a signification main effect of Time, *F* (1, 33) = 11.00, *p* < .01, *η*_p_^2^ = .25, and Group x Time interaction, *F* (2, 33) = 6.53, *p* < .01, *η*_p_^2^ = .28, were observed. Follow-up paired sample t tests were conducted for each group separately. This revealed that the MBP group showed a significant increase of positive automatic thoughts over time, but this tendency was not observed in other groups, namely the CRP and the Control(MBP: *t* [11] = -3.79, *p* < .01, *d* = -1.20; CPR: *t* [11] = -.87, *p* = .41, *d* = -0.13; Control: *t* [11] = -.13, *p* = .90, *d* = -0.01). Lastly, in the results for PANAS, the time effect was not significant, *F* (1, 33) = .67, *p* = .42, *η*_p_^2^ = .02, nor was the Time × Group interaction, *F* (2, 33) = .67, *p* = .52, *η*_p_^2^ = .04. The changes of the RTA and ATQ-P among groups between time points presented in Fig 2.

**Table 1:** Test anxiety, positive thoughts and affect mean scores pre- and post-group treatment.

	MBP *M(SD)*		CRP *M(SD)*		Control *M(SD)*		Group	Time	Time × Group
	Pre	Post	Pre	Post	Pre	Post	*F*	*F*	*F*
RTA	49.75(5.71)	38.58(10.04)	50.58(6.26)	41.17(8.94)	47.92(7.74)	43.33(8.49)	.19	57.05***	3.14^+^
ATQ-P	86.67(13.77)	100.83(9.80)	87.58(16.69)	90.00(19.46)	89.42(20.17)	89.67(17.67)	.34	11.00**	6.53**
PANAS	63.83(9.78)	66.50(8.49)	60.92(5.82)	62.75(7.76)	63.83(7.08)	62.75(8.82)	.70	.67	.67

^+^*p* = .056

**p < .01

***p < .001.

M = mean; SD = standard deviation; MBP = Mindful Breathing Practice; CRP = Cognitive Reappraisal Practice; pre = pre-assessment; post = post-assessment; RTA = Revised Test Anxiety scale; ATQ-P = Automatic Thoughts Questionnaire-Positive scale; PANAS = Positive And Negative Affect Schedule.

## Discussion

This study examined the effect of daily mindfulness breathing practices on test anxiety for highly anxious students. At this end, the effect of mindful breathing practice was compared to those of cognitive reappraisal practice and no-training control. The present study showed two key findings. Firstly, both mindfulness breathing and cognitive reappraisal practices yielded a large effect size in reducing test anxiety for undergraduate students. Secondly, mindfulness breathing practice showed increased positive automatic thoughts over time, compared to their counterparts, the cognitive reappraisal practice and control group. Our findings showed that the effects of the MBP on test anxiety were comparable to those of the CRP. This is consistent with the results of the previous study that suggested that mindfulness or cognitive reappraisal could be equally effective on reducing test anxiety (e.g., [28]). Hayes-Skelton and Graham [29] indicated that there might be decentering in common between the MBP and CRP; the relationship between mindfulness and social anxiety was partially accounted for by decentering, whereas the relationship between cognitive reappraisal and social anxiety is more fully accounted for by decentering. The decentering enables people to distance and disidentify themselves from the contents of their conscious thoughts and emotions [30]. By this, they gain a sense of mastery over their thoughts and emotions and feel able to perceive them as transient mental events, rather than to identify with them or to believe that thoughts and emotions are accurate reflections of the self or reality. Therefore, decentering aids in disengaging from self-criticism, rumination, and anxiety that can arise when reacting to negative thinking patterns [31].

In this context, we speculated that decentering components of the MBP and CRP might contribute to reduce test anxiety. To confirm this possibility, future studies should identify specific therapeutic components of the MBP and CRP related to text anxiety. In addition, this study was designed in a way that required the participants to practice daily mindful breathing outside the lab for a period of one week after taking one session of education. This self-training helped the participants improve their capacities for self-regulation, which in turn may have helped them deal more effectively with test anxiety. The no-training condition showed a decreased test anxiety; however, the effect size of the no-training condition was smaller than those of the MBP and CRP. There might be due to a process of natural change over time as shown in Shikantani’s study [28]. Another possibility might be compensation for the participation of the study. Participants who expected to receive a gift coupon at the end of study might have felt pressure to contribute to the results of the study as they already knew the aim of the study. Selection bias at the screening stage might also have affected the results of the no-training group. For example, although they were not highly anxious, they might have been interested in participating in the study due to compensation for the study. If so, the lower scores of test anxiety shown in the end of the study might reflect their real anxiety level.

The results of the current study indicated that the practice of MBIs may be more effective than cognitive appraisal in helping students to deal with positive thinking. This means that it may be advantageous to approach the testing situation with an accepting and nonjudgmental mindset rather than to teach students to engage with their fearful cognitions through labeling and disputation. A cognitive dimension of test anxiety is to worry about the personal and social consequences of failing to obtain one’s performance goals from the testing situation (e.g. test-irrelevant thinking) [32]. However, if people who have test anxiety practice mindfulness to focus on the present moment, this can free their minds from worrying and focusing on future events. Also, mindfulness may facilitative effects on cognitive switching because it makes people increase their ability to shift their focus from one object to the next [33]. Mindfulness facilitates the attribution of new meaning to previously stressful events, thus it can stimulate positive thoughts to break away from negative thinking like worrying about tests. In the same vein, Garland et al [34] suggested that mindfulness, as a metacognitive form of awareness, involves the process of decentering, which is a shifting of cognitive sets that enables alternate appraisals of life events(positive reappraisal). In this study, mindfulness practices could be making participants take a cognitive shift and increase their positive thinking.

In addition, participants wrote about what happened in their body and mind for the mindful practice on their daily sheet. In contrast to writing about cognitive reappraisals that focused on changing their negative thinking, writing about mindfulness encouraged participants to observe their emotions and thoughts objectively. Participants could notice their experience on not only negative thinking but also neutral thinking, which could promote getting a whole range of perspectives on their experiences through metacognition. Thus, the writing experiences for mindful practices might be more valuable for getting a new perspective on its own than mindful practices itself. This finding agrees with the belief that mindfulness practice reduces the tendency to overlook novel solutions to a situation due to rigid and repetitive thought patterns formed through experience [35].

Individuals with test anxiety may have any one of several physical, mental, emotional or pragmatic obstacles in seeking treatment, such as the cost of treatment, loss of time required, the distance to available treatment and emotional discomfort associated with the stigma. Developing self-directed training programs based on MBI will increase treatment accessibility for those who suffer from test anxiety wherever they live. Based on our study results, we suggest that an ongoing program of MBP could become a foundation of resilience for participants (from a neuropsychological point of view). Future studies of the efficacy of MBP should be conducted with follow-up assessment to determine the duration of MBP`s positive and beneficial effects. In addition, research into other mindfulness based training should be undertaken to explore other potential adaptive strategies for coping with test anxiety. This study has several strengths. First, it compared the effectiveness of two prospective strategies (MBP and CRP), when used as treatment interventions for test anxious individuals. Second is the fact that these interventions were not implemented at a single session but at multiple sessions (one session of education and six sessions by themselves). Third, recording experience for daily mindful practice could be helping people with anxiety improve their positive thinking. Also, researcher confirmed whether participants did mindfulness or not to encourage them keep going on that might be able to induce them self-training and self-regulation. Along with its obvious strengths, this study had several weaknesses. First of all, the sample size was relatively small, so the results of this study couldn’t generalize to people with test anxiety. Also, the study couldn’t avoid sampling biases because participants knew that some of the respondents could be recruited for an experimental study. However, even though it could raise the sampling bias, we felt that participants should be aware of what they would be asked to do. Secondly, the recruitment and selection of study participants was largely dependent upon their self-reporting as a measure of test anxiety. In future studies, it will be important to consider obtaining a larger sample size and getting psychophysiological measures to improve objectivity. Thirdly, we introduced students with high test anxiety to daily mindful practices and helped them reduce test anxiety in a potential evaluation environment, but we didn’t intervene in a real testing situation. Therefore, in the future, rigorous criteria should be used when recruiting participants who identify as having a history of test anxiety and this intervention could be applied in a real test situation.

## Supporting Information

S1 Data Data file. (SAV)

S1 Questionnaires Questionnaire file. (DOCX)
