# Supplementary material for: The Effectiveness of Daily Mindful Breathing Practices on Test Anxiety of Students
Source: PLoS One. 2016 Oct 20;11(10):e0164822. doi: 10.1371/journal.pone.0164822 (PMC5072593; doi:10.1371/journal.pone.0164822)
Supplement: S1 Questionnaires — (DOCX) [file pone.0164822.s002.docx]

RTA

The following items refer to how you feel when taking a test. Use the scale below to rate Items 1 through 20 in terms of how you feel when taking tests in general.

| Item | | Almost never | Sometimes | Often | Almost always |
| --- | --- | --- | --- | --- | --- |
| 1 | Thinking about my grade in a course interferes with my work on tests. | 1 | 2 | 3 | 4 |
| 2 | I seem to defeat myself while taking important tests. | 1 | 2 | 3 | 4 |
| 3 | During tests I find myself thinking about the consequences of failing. | 1 | 2 | 3 | 4 |
| 4 | I start feeling very uneasy just before getting a test paper back. | 1 | 2 | 3 | 4 |
| 5 | During tests I feel very tense. | 1 | 2 | 3 | 4 |
| 6 | I worry a great deal before taking an important exam. | 1 | 2 | 3 | 4 |
| 7 | During tests I find myself thinking of things unrelated to the material being tested. | 1 | 2 | 3 | 4 |
| 8 | While taking tests, I find myself thinking how much brighter the other people are. | 1 | 2 | 3 | 4 |
| 9 | I think about current events during a test. | 1 | 2 | 3 | 4 |
| 10 | I get a headache during an important test. | 1 | 2 | 3 | 4 |
| 11 | While taking a test, I often think about how difficult it is. | 1 | 2 | 3 | 4 |
| 12 | I am anxious about tests. | 1 | 2 | 3 | 4 |
| 13 | While taking tests I sometimes think about being somewhere else. | 1 | 2 | 3 | 4 |
| 14 | During tests I find I am distracted by thoughts of upcoming events. | 1 | 2 | 3 | 4 |
| 15 | My mouth feels dry during a test. | 1 | 2 | 3 | 4 |
| 16 | I sometimes find myself trembling before or during test. | 1 | 2 | 3 | 4 |
| 17 | While taking a test my muscles are very tight. | 1 | 2 | 3 | 4 |
| 18 | I have difficulty breathing while taking a test. | 1 | 2 | 3 | 4 |
| 19 | During the test I think about how I should have prepared for the test. | 1 | 2 | 3 | 4 |
| 20 | I worry before the test because I do not know what to expect. | 1 | 2 | 3 | 4 |

ATQ-P

Please read each statement below and rate how often you think about following statements during the past week.

| Item | | Never | Rarely | Some-times | Often | Always |
| --- | --- | --- | --- | --- | --- | --- |
| 1 | I am respected by my peers. | 1 | 2 | 3 | 4 | 5 |
| 2 | I have a good sense of humor. | 1 | 2 | 3 | 4 | 5 |
| 3 | My future looks bright. | 1 | 2 | 3 | 4 | 5 |
| 4 | I will be successful. | 1 | 2 | 3 | 4 | 5 |
| 5 | I’m fun to be with. | 1 | 2 | 3 | 4 | 5 |
| 6 | I am in a great mood. | 1 | 2 | 3 | 4 | 5 |
| 7 | There are many people who care about me. | 1 | 2 | 3 | 4 | 5 |
| 8 | I’m proud of my accomplishment. | 1 | 2 | 3 | 4 | 5 |
| 9 | I will finish what I start. | 1 | 2 | 3 | 4 | 5 |
| 10 | I have many good qualities. | 1 | 2 | 3 | 4 | 5 |
| 11 | I am comfortable with life. | 1 | 2 | 3 | 4 | 5 |
| 12 | I have a good way with others. | 1 | 2 | 3 | 4 | 5 |
| 13 | I am a lucky person. | 1 | 2 | 3 | 4 | 5 |
| 14 | I have friends who support me. | 1 | 2 | 3 | 4 | 5 |
| 15 | Life is exciting. | 1 | 2 | 3 | 4 | 5 |
| 16 | I enjoy a challenge. | 1 | 2 | 3 | 4 | 5 |
| 17 | My social life is terrific. | 1 | 2 | 3 | 4 | 5 |
| 18 | There’s nothing to worry about. | 1 | 2 | 3 | 4 | 5 |
| 19 | I’m so relaxed. | 1 | 2 | 3 | 4 | 5 |
| 20 | My life is running smoothly. | 1 | 2 | 3 | 4 | 5 |
| 21 | I’m happy with the way I look. | 1 | 2 | 3 | 4 | 5 |
| 22 | I take good care of myself. | 1 | 2 | 3 | 4 | 5 |
| 23 | I deserve the best in life. | 1 | 2 | 3 | 4 | 5 |
| 24 | Bad days are rare. | 1 | 2 | 3 | 4 | 5 |
| 25 | I have many useful qualities. | 1 | 2 | 3 | 4 | 5 |
| 26 | There is no problem that is hopeless. | 1 | 2 | 3 | 4 | 5 |
| 27 | I won’t give up. | 1 | 2 | 3 | 4 | 5 |
| 28 | I state my opinions with confidence. | 1 | 2 | 3 | 4 | 5 |
| 29 | My life keeps getting better. | 1 | 2 | 3 | 4 | 5 |
| 30 | Today I’ve accomplished a lot. | 1 | 2 | 3 | 4 | 5 |

PANAS

This scale consist of a number of words that describe different feelings and emotions. Read each item and then mark the appropriate answer in the space next to that word. Indicate to what extent you have felt this way during the past week.

| Item | | Very slightly or not at all | A little | Moderately | Quite a bit | Extremely |
| --- | --- | --- | --- | --- | --- | --- |
| 1 | Interested | 1 | 2 | 3 | 4 | 5 |
| 2 | Irritable | 1 | 2 | 3 | 4 | 5 |
| 3 | Distressed | 1 | 2 | 3 | 4 | 5 |
| 4 | Alert | 1 | 2 | 3 | 4 | 5 |
| 5 | Excited | 1 | 2 | 3 | 4 | 5 |
| 6 | Ashamed | 1 | 2 | 3 | 4 | 5 |
| 7 | Upset | 1 | 2 | 3 | 4 | 5 |
| 8 | Inspired | 1 | 2 | 3 | 4 | 5 |
| 9 | Strong | 1 | 2 | 3 | 4 | 5 |
| 10 | Nervous | 1 | 2 | 3 | 4 | 5 |
| 11 | Guilty | 1 | 2 | 3 | 4 | 5 |
| 12 | Determined | 1 | 2 | 3 | 4 | 5 |
| 13 | Scared | 1 | 2 | 3 | 4 | 5 |
| 14 | Attentive | 1 | 2 | 3 | 4 | 5 |
| 15 | Hostile | 1 | 2 | 3 | 4 | 5 |
| 16 | Jittery | 1 | 2 | 3 | 4 | 5 |
| 17 | Enthusiastic | 1 | 2 | 3 | 4 | 5 |
| 18 | Active | 1 | 2 | 3 | 4 | 5 |
| 19 | Proud | 1 | 2 | 3 | 4 | 5 |
| 20 | Afraid | 1 | 2 | 3 | 4 | 5 |
